# A Clinical Study of Acquired Immunodeficiency Syndrome Associated *Penicillium Marneffei* Infection from a Non-Endemic Area in China

**DOI:** 10.1371/journal.pone.0130376

**Published:** 2015-06-17

**Authors:** Jinding Zheng, Xien Gui, Qian Cao, Rongrong Yang, Yajun Yan, Liping Deng, Jonathan Lio

**Affiliations:** 1 Department of Infectious Diseases, Zhongnan Hospital of Wuhan University, Wuhan, Hubei, China; 2 Department of Medicine, University of Chicago, Chicago, Illinois, United States of America; The University of Wisconsin - Madison, UNITED STATES

## Abstract

**Objective:**

To investigate the clinical characteristics, diagnosis, treatment and prognosis of penicilliosis among the patients with acquired immunodeficiency syndrome (AIDS) in non-endemic areas of China, and then to discuss its incubation period and the diagnostic performance of serum galactomannan test for penicilliosis.

**Methods:**

Medical records and travel histories of penicilliosis patients in Zhongnan hospital from January 2006 to December 2013, and the interval from when the patients left the endemic area to the onset of the disease was analyzed. Serum galactomannan levels of penicilliosis patients and AIDS patients with fever were measured by the Platelia Aspergillus Enzyme Immunoassay Kit.

**Results:**

A total of 47 AIDS-associated penicilliosis were confirmed by fungal culture, which accounted for 4.8% of 981 AIDS-related admissions. The sensitivity and specificity of serum galactomannan test for penicilliosis were 95.8% (23/24) and 90.9% (30/33), respectively, (cutoff index = 1.0). Two independent predictors for early mortality (death within 12 weeks) of the patients (21.3%, 10/47) were a delayed diagnosis and no treatment with antifungal therapy. Among 14 patients who became ill after leaving endemic areas, ten patients presented with the onset symptoms within 12 months (from 11 days to 360 days). We found a patient living with asymptomatic *P*. *marneffei* fungemia who had not received any antifungal therapy until 18 months’ follow up.

**Conclusions:**

The co-infection of *P*. *marneffei* and HIV was not uncommon in the non-endemic areas of penicilliosis in China. There exists a latent form of infection for *P*. *marneffei*. The incubation period of penicilliosis may be quite different from one patient to another. In AIDS patients, the serum galactomannan test has utility for the diagnosis of penicilliosis. When patients with penicilliosis/AIDS were diagnosed early and treated with standardized antifungal therapy and combined antiretroviral therapy, their prognosis improved.

## Introduction

Penicilliosis, a systemic mycosis caused by *Penicillium marneffei* and an important endemic fungus in Southeast Asia, is known to be a common opportunistic infection in patients with acquired immunodeficiency syndrome (AIDS). In these areas, about 50,000 human immunodeficiency virus (HIV) positive patients are newly infected by *P*. *marneffei* each year and which results in up to 5,000 deaths annually[1]. However, sporadic cases of penicilliosis has been observed in immunocompromised individuals from non-endemic countries such as the Unites States, Japan, Korea and Germany, who have traveled to the endemic areas[2–5]. In mainland China, penicilliosis is endemic in the southern provinces such as Guangxi, Guangdong, Fujian, Yunnan, Hunan, and Hong Kong[6, 7], but there have been no case reports of penicilliosis from other regions of China.

In the past 30 years, due to imbalanced economic development, a large number of workers have migrated from northern to southern China. Some of them have been co-infected with HIV and *P*. *marneffei* and have gone back to their home provinces with these pathogens several years after. However, many healthcare workers in non-endemic areas are not aware of this phenomenon, which has sometimes led to delayed diagnosis or misdiagnosis. Therefore, the aim of this study is to draw the clinician’s attention to this disease by presenting the clinical characteristics, diagnosis, treatment and prognosis of penicilliosis in a series of HIV positive patients from a non-endemic area of China (Hubei province).

## Materials and Methods

### Study population and data collection

Zhongnan Hospital of Wuhan University is the largest referral hospital for HIV/AIDS care in Hubei Province. From January 2006 to December 2013, medical records of patients who were co-infected by HIV and *P*. *marneffei* were collected and their traveling histories to the endemic areas were queried by a face-to-face interview. Data collected included the age, gender and CD4+T lymphocyte count of the patients, the clinical presentation of penicilliosis, associated laboratory examinations, chest computed tomography (CT) scans, and the outcomes of the patients. Penicilliosis was diagnosed once *P*. *marneffei* has been isolated from the blood, bone marrow, lymph nodes, sputum, skin scrapings, or broncho-alveolar lavage fluid (BAL) samples, according to standard culture techniques[8]. This clinical study was conducted according to the principles expressed in the Declaration of Helsinki. The Ethical approval was granted by the Ethics Committee of Zhongnan Hospital of Wuhan University(NO.201501). Written consent forms were obtained from the patients after gave them appropriate information.

### Serum samples

Serum samples were obtained from the penicilliosis patients before treatment with amphotericin B (AMB). Serum samples of the controls were obtained from AIDS patients with fever but without penicilliosis, which was excluded by negative cultures of the blood and marrow. Once obtained, all the serum samples were stored in sterile tubes at -70°C immediately.

### Definition of outcomes

The outcome of the patients was categorized as follows after one year’s follow up: 1) sustained response: after standard treatment with AMB, the culture for *P*. *marneffei* became negative and the penicilliosis-related symptoms and signs resolved for at least 1 year; 2) relapse: the culture for *P*. *marneffei* became negative and the penicilliosis-related symptoms and signs resolved after treatment, but recurred within a year; 3) death.

### Galactomannan (GM) antigen detection

Serum GM levels were determined using the Platelia Aspergillus Enzyme Immunoassay Kit following the manufacturer’s instructions (Bio-Rad, Marnes-la-Coquette, France).

### The interval from the patients leaving endemic areas to onset of penicilliosis

**Fig 1:**
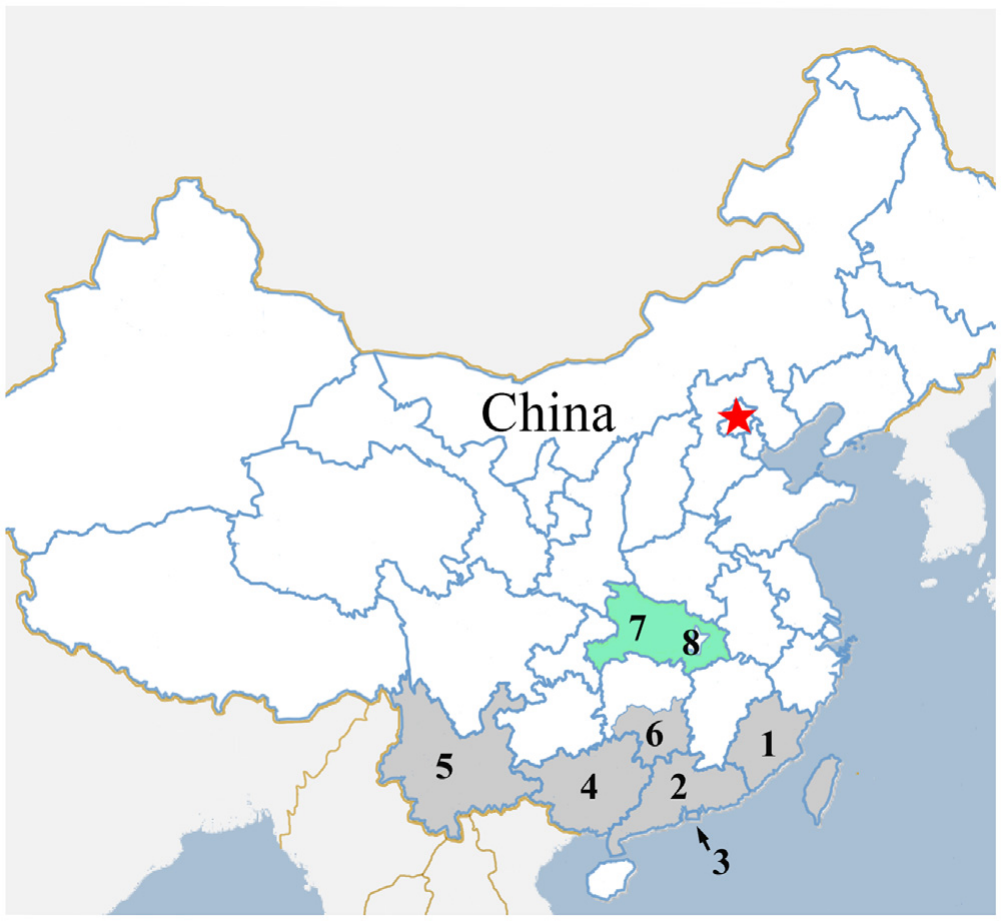
The endemic regions of penicilliosis in China. The red five-pointed star represents the location of Beijing, the capital of China. The green area in the map is Hubei Province (7) with Wuhan City (8) as its capital. The gray areas in the map are the endemic regions for penicilliosis in China, 1.Fujian(4 patients), 2.Guangdong(36 patients), 3. Hong Kong(0 patient), 4.Guangxi(3 patients), 5.Yunnan(1 patient), 6.Southern Hunan(3 patients).

Despite the fact that the transmission mode of *P*. *marneffei* infection is unknown, contact with a penicilliosis endemic region is a known key risk factor. We investigated the interval from the patients leaving endemic areas to onset of penicilliosis for estimating the incubation period of *P*. *marneffei*. Following a literature review (searching PubMed Database, China National Knowledge Infrastructure and Wanfang Database from 1980 to 2013) and consultation with Center for Disease Control and Prevention in some regions, endemic regions for penicilliosis within China were defined and mapped (Fig 1). The onset time was defined as the time of occurrence for penicilliosis-related symptoms and signs such as fever, cough, sputum production, cutaneous lesions, hepatosplenomegaly or lymphadenopathy. The incubation period was defined as the interval between the time when the patients left the endemic area and the onset time. All patients who had fallen ill and had been diagnosed for penicilliosis before leaving the endemic area have been excluded from our study. If a patient had repeatedly visited the endemic areas, then the last time of leaving was used for calculation.

### Statistical analysis

Normally distributed continuous variables were described as mean±standard deviation, non-normally distributed continuous variables described as median and interquartile range (IQR). We performed univariate and multivariate logistic regressions by forward stepwise to identify risk factors for all-cause mortality at 12 weeks. Statistically significant results (*P* < 0.10) from the univariate analysis were selected for analysis by multivariate logistic regression. Statistical analyses were performed using SPSS16.0 (SPSS Inc., Chicago, IL, USA). Differences were considered significant when *P*-values were < 0.05.

## Results

### Prevalence of *P*. *marneffei* infection among patients with AIDS

During January 2006 to December 2013, 47 inpatients out of a total number of 981 HIV-related hospitalization (4.8%) in Zhongnan Hospital were confirmed for *P*. *marneffei* infection by fungal culture. No patients with penicilliosis were diagnosed in the outpatient setting. All the 47 patients were born in Hubei Province and had worked or shortly stayed in the endemic areas of *P*. *marneffei* in southern China. The endemic areas which the patients had visited are noted in Fig 1. The medium age of the patients was 32 years old (IQR:26–40 years) with 29 (61.7%) male patients and 18 (38.3%) female patients.

In addition, only 3 (3/981, 3.1‰) patients were confirmed for aspergillosis by biopsy and culture among 981 HIV-infected patients; twenty-six (26/981, 2.7%) patients out of 981 AIDS-related admissions died during hospitalization, among them, 3 deaths (3/26, 11.5%) were caused by penicilliosis, just behind cryptococcosis (8/26, 30.8%) and pneumocystosis (6/26, 23.1%).

### Clinical features of patients with penicilliosis

The clinical and radiological features of the patients are shown in Tables 1 and 2. The most common symptoms were fever (40/47, 85.1%), followed by respiratory symptoms (28/47, 59.6%), skin lesions (27/47, 57.4%), digestive symptoms (23/47, 48.9%), hepatosplenomegaly (32/47, 68.1%), and lymphadenopathy (10/47, 21.3%). Forty-four out of 47 patients had a chest CT scan, among them, 37 patients (37/44, 84.1%) had one or more abnormal characteristics including pleural effusion, alveolar consolidation, nodule lesions, miliary lesions, and cavitary lesions. Concurrent opportunistic infections were diagnosed in 11 (11/47, 23.4%) of 47 patients, including 3 patients confirmed with *Pneumocystis jirovecii* pneumonia (PCP) by bronchoalveolar lavage (BAL) or/and sputum specimens with Gomori methenamine silver, 2 patients diagnosed with cytomegalovirus retinitis by ophthalmoscopic examination, 2 patients confirmed with tuberculosis by microscopy and/or culture, 1 patient confirmed with pulmonary mucormycosis by BAL culture, 1 patient confirmed with co-infection with septicemia of *Escherichia coli* and *Nocardia* by blood culture, 1 patient co-infected with PCP and tuberculosis,and 1 patient co-infected with tuberculosis and cytomegalovirus retinitis.

**Table 1:** Factors associated with death within 12 weeks of penicilliosis patients.

Variables^1^	Penicilliosis patients N = 47	Survival in 12 weeks N = 37	Death in 12 weeks N = 10	Univariate effectc: OR (95% CI)	Multiple logistic regressiond: OR (95% CI)
**Sex**					
**Male**	61.7%(29/47)	59.5%(22/37)	70.0%(7/10)	1.591(0.354–7.154),*P* = 0.411	
**Female**	38.3%(18/47)	40.5%(15/37)	30.0%(3/10)	0.629(0.140–2.826),*P* = 0.411	
**Age median (IQR)**	32(26–40)	31(26–40)	36(29–41)	1.014(0.941–1.093),*P* = 0.713	
**Newly diagnosed HIV patients**	**72.3%(34/47)**	**78.4%(29/37)**	**50.0%(5/10)**	**0.276(0.064–1.195),*P* = 0.087**	
**The days from admission to diagnosis median (range)**	6(3–37)	6(3–22)	7(3–37)	1.086(0.974–1.211),*P* = 0.138	
**The days from onset to diagnosis median (range)**	**36(7–212)**	**32(7–187)**	**81(25–212)**	**1.023(1.006–1.040),*P* = 0.009**	**1.019(1.000–1.038),*P* = 0.046**
**cART**	17.0%(8/47)	13.5%(5/37)	30.0%(3/10)	2.743(0.528–14.261),*P* = 0.217	
**Symptoms/signs**					
**Oropharyngeal pseudomembranous lesions**	44.7%(21/47)	45.9%(17/37)	40.0%(4/10)	0.784(0.189–3.247),*P* = 0.512	
**Fever** ^**2**^	85.1%(40/47)	81.1%(30/37)	100.0%(10/10)	NS,*P* = 0.164	
**Skin lesions**	57.4%(27/47)	59.5%(22/37)	50.0%(5/10)	0.628(0.168–2.772),*P* = 0.426	
**Respiratory symptoms** ^**3**^	59.6%(28/47)	59.5%(22/37)	60.0%(6/10)	1.023(0.246–4.253),*P* = 0.634	
**Digestive symptoms** ^**4**^	48.9%(23/47)	43.2%(16/37)	70.0%(7/10)	3.062(0.683–13.736),*P* = 0.126	
**Hepatosplenomegaly**	68.1%(32/47)	70.3%(26/37)	60.0%(6/10)	0.635(0.149–2.701),*P* = 0.397	
**Lymphadenopathy**	21.3%(10/47)	21.6%(8/37)	20.0%(2/10)	0.906(0.160–5.142),*P* = 0.643	
**Laboratory findings**					
**CD4 cells/μL median (IQR)**	8(3–21)	8(4–22)	9(3–22)	1.002(0.975–1.029),*P* = 0.900	
**Platelets<100×10^9^ cells/L**	61.7%(29/47)	56.8%(21/37)	80.0%(8/10)	3.048(0.568–16.360),*P* = 0.166	
**Neutropenia (Neutrophils<0.5×10^9^ cells/L)**	0.0%(0/47)	0.0%(0/37)	0.0%(0/10)	NS^5^	
**Agranulocytosis (Neutrophils<2.0×10^9^ cells/L)**	25.5%(12/47)	24.3%(9/37)	30.0%(3/10)	1.333(0.284–6.263),*P* = 0.501	
**ALT>46 U/L**	40.4%(19/47)	37.8%(14/37)	50.0%(5/10)	1.643(0.403–6.705),*P* = 0.366	
**AST>46 U/L** ^**6**^	**74.5%(35/47)**	**67.6%(25/37)**	**100.0%(10/10)**	**NS,*P* = 0.035**	
**BUN>7.2 μmol/L**	**17.0%(8/47)**	**8.1%(3/37)**	**50.0%(5/10)**	**11.333(2.046–62.771),*P* = 0.007**	
**Cr>100 μmol/L**	**17.0%(8/47)**	**10.8%(4/37)**	**40.0%(4/10)**	**5.500(1.071–28.248),*P* = 0.051**	
**Other concurrent opportunistic infections** ^**7**^	23.4%(11/47)	24.3% (9/37)	20.0% (2/10)	0.778(0.139–4.352), *P* = 0.570	
**No treatment with antifungal therapy**	**10.6%(5/47)**	**2.7%(1/37)**	**40.0%(4/10)**	**24.000(2.276–253.063),*P* = 0.005**	**14.178(1.084–185.382),*P* = 0.043**

^1^ The clinical features and laboratory data of patients are from the first examination upon admission to hospital. IQR, interquartile range; cART, combined antiretroviral therapy; ALT, alanine aminotransferase; AST, aspartate aminotransferase; BUN, blood urea nitrogen; Cr, creatinine.

^2,6^ We could not calculate the OR value and the factors were not entered into the multivariate analysis since the frequency was zero.

^3^ Respiratory symptoms: including cough, sputum production, dyspnea.

^4^ Digestive symptoms: including nausea, vomiting, abdominal pain, diarrhea.

^5^ NS: No statistics.

^7^ Other concurrent opportunistic infections including: *Pneumocystis jirovecii* pneumonia, cytomegalovirus retinitis, tuberculosis, pulmonary mucormycosis, septicemia of *Escherichia coli* and *Nocardia*.

**Table 2:** Chest CT scan findings of 44 penicilliosis patients.

Chest CT scan findings	Abnormality/examination(%)
**Pleural effussion**	18/44(40.9)
**Alveolar consolidation**	15/44(34.1)
**Nodule lesions**	12/44(27.3)
**Miliary lesions**	8/44(18.2)
**Cavitary lesions**	2/44(4.5)
**Total**	37/44(84.1)

### Laboratory findings

*P*. *marneffei* was isolated successfully from the bone marrow (31/38, 81.6%), blood (36/46, 78.3%), BAL (8/9, 88.9%), sputum (11/17,64.7%), stool (10/16, 62.5%), urine (7/9, 77.7%), skin biopsy/scrapings (8/8), ascites (2/2), lymph node (2/2), and ocular humor (1/1) of the patients, except for 8 cerebrospinal fluid samples from which no *P*. *marneffei* was found.

Twelve patients out of 47 had neutropenia (<2.0×10^9^cells/L), but none of these had agranulocytosis (<0.5×10^9^cells/L). All of the 47 patients had a CD4+ T-cell count <200 cells/ul, with a median of 8cells/μL (IQR: 3–21 cells/μL). Forty-four patients had a CD4+ T-cell count ≤50 cells/μL (44/47, 93.6%); 2 patients had a CD4+ T-cell count between 51 to 100 cells/μL (2/47, 4.3%); just one patient had a CD4+ T-cell count ≥100 cells/μL (1/47, 2.1%). (Table 1)

Nineteen (40.4%) out of 47 patients had an abnormal alanine aminotransferase (ALT) level (>46U/L), with a median level as 94 U/L (IQR: 58–116 U/L); 35 (74.5%) patients had an abnormal aspartate aminotransferase (AST) level (>46 U/L), with a median level as 150 U/L (IQR: 78–305 U/L); 8 (17.0%) patients had an abnormal blood urea nitrogen (BUN) level (>7.2 μmol/L), with a median level as 14.0 μmol/L (IQR:10.7–18.9 μmol/L); 8(17.0%) patients had an abnormal creatinine (Cr) (>100 μmol/L), with a median level as 133 μmol/L (IQR: 112–259 μmol/L). (Table 1)

### Diagnosis timing of AIDS and penicilliosis

Thirty-four patients out of 47 (72.3%) were hospitalized at first with penicilliosis and who were diagnosed as positive for HIV infection afterward. The rest of the patients (13/47, 27.7%) had been diagnosed with HIV before admission, and 8 (8/13, 61.5%) patients were taking combined antiretroviral therapy (cART). The median interval from the onset of penicilliosis symptoms to the confirmed diagnosis was 36 days (range: 7–212 days), and the median interval from the time of admission to our hospital to the diagnosis of penicilliosis was 6 days (range: 3–37 days). (Table 1)

### Serum GM antigen detection

Since the utility of the galactomannan(GM) test for diagnosis of penicilliosis in AIDS patients was unknown, we compared GM antigen levels between 24 AIDS patients with penicilliosis (the GM test was only done on 24 of the 47 patients with penicilliosis because the other 23 patients refused blood draw for it.) and 33 AIDS patients with other OIs. The CD4+ T cell median counts of 24 patients with penicilliosis was 6 cells/μL (range:1–61 cells/μL), and the CD4+ T cell median counts of 33 patients with the other OIs was 12 cells/μL (range:1–80 cells/μL); the CD4+ T cell median counts were not significantly different between the patients with penicilliosis and the patients with other OIs (*p* = 0.093, Mann-Whitney U test). Of the 33 patients with other OIs, 21 patients were infected with *Cryptococcus neoformans* confirmed by cultures of clinical specimens including blood, bone marrow and cerebrospinal fluid; 5 patients infected were with *Mycobacterium tuberculosis* confirmed by culture of clinical specimens including BAL and sputum; 3 patients were infected with *Pneumocystis jiroveci pneumonia* confirmed by BAL with Gomorimethenamine silver; 2 patients were infected with cytomegalovirus retinitis confirmed by ophthalmoscopic examination and PCR of vitreous for cytomegalovirus; 1 patient was fungemic with *Candida albicans*; 1 patient was fungemic with *Candida tropicalis*.

The sensitivity and specificity of serum GM testing for penicilliosis were 95.8% (23/24) and 90.9% (30/33) respectively, (cutoff index = 1.0), as shown in Table 3. It took about 3 hours to determine serum GM antigen by Platelia Aspergillus Enzyme Immunoassay Kit. The culture diagnosis median time of 23 penicilliosis patients with positive GM results was 7 days (range: 3–17 days) after their admission. If serum GM antigen was obtained the day after admission, it would be 6 days earlier than the fungal culture for diagnosis of penicilliosis.

**Table 3:** CD4+ T cell count and GM test positive rate of patients with penicilliosis and without penicilliosis.

	CD4+ T cell count	GM test		
Groups	Median(range) cells/μL	Median(range)	Positive No.	Positive rate
**Penicilliosis group (n = 24)** ^**1**^	6(1–61)	5.354 (0.784–6.428)	23	95.80%
** Fungemia (n = 17)**	4(1–14)	5.335 (1.782–6.428)	17	100.00%
** Without fungemia (n = 7)**	22(1–61)	5.462(0.784–6.336)	6	85.70%
**Without penicilliosis group (n = 33)**	12(1–80)	0.422(0.247–2.521)	3	9.10%
** Cryptococcosis (n = 21)** ^**2**^	9(1–54)	0.443 (0.277–2.521)	2	9.52%
** The other OIs patients (n = 12)** ^**3**^	14(1–80)	0.359 (0.247–2.232)	1	8.33%

^1,2^ The serum samples were obtained before treated with amphotericin B.

^3^ OIs: opportunistic infections.

There were 2 patients with cryptococcosis and 1 patient with tuberculosis with positive GM results. Of the three patients, one cryptococcosis patient had a halo sign by chest CT scan and tissue biopsy was not performed; the other two patients had no evidence of a halo sign, air crescent sign, or cavities on chest CT, which are typical signs of aspergillosis. The 2 cryptococcosis patients were treated with AMB, one patient died within 12 weeks after antifungal therapy, the other patient with a halo sign survived at 1 year follow up; the tuberculosis patient was dead within 1 year after anti-tuberculosis treatment (without antifungal therapy).

### Treatment and prognosis of penicilliosis patients

Once the patients were diagnosed with penicilliosis, intravenous AMB (0.6 mg/kg) was started for 2 weeks’ treatment followed by oral itraconazole at a dose of 200 mg twice daily for 10 weeks; once the temperature of the patients returned to normal with significant improvement of their clinical symptoms, cART was started for the AIDS patients, usually two weeks after the beginning of AMB treatment. Itraconazole was continued at 200 mg once daily to prevent relapse until the patients regained a CD4+ T-cell count >100/μL for over 6 months[9].

All 47 penicilliosis patients were followed up for 1 year after the diagnosis of penicilliosis. The all-cause mortality rate within 12 weeks was 21.3% (10/47) and within 1 year was 27.7% (13/47). Among the 13 patients who died within 1 year, 9 (19.1%, 9/47) deaths were due to penicilliosis, 1 death was due to the co-infection of *P*. *marneffei*/*Pneumocystis jirovecii*/*M*. *tuberculosis*, 1 death was due to the co-infection of *P*. *marneffei*/*Escherichia coli*/*Nocardia*, 1 death was due to the co-infection of *P*. *marneffei*/*M*. *tuberculosis*, and 1 death was due to cytomegalovirus infection. Among these 47 patients, 5 patients did not receive the antifungal therapy, and 4 (80.0%, 4/5) of them died within 12 weeks; of the other 42 patients who have received the antifungal therapies, 6 (14.3%, 6/42) patients died within 12 weeks, and 3 patients died within 12 weeks to 1 year after the diagnosis of penicilliosis. As for the 34 patients who survived, 2 (5.9%, 2/34) of them relapsed due to improper withdrawal of the antifungal therapies when their CD4+ T-cell count reached 46cells/μL and 37cells/μL. After relapse, *P*. *marneffei* was again isolated from their sputum and lymph node respectively, and re-treatment with AMB and itraconazole was efficacious.

Univariate analysis showed significantly higher risk of early death (within 12 weeks) among penicilliosis patients (10 vs. 47), and the other risk factors were AST>46U/L, BUN>7.2 μmol/L, Cr>100 μmol/L, the length of time from onset to diagnosis, and the absence of antifungal treatment. However, after multivariate analysis, only the length of time from onset to diagnosis (OR 1.019, CI 95%:1.000–1.038, *P* = 0.046) and the absence of antifungal treatment (OR 14.178, CI 95%:1.084–185.382, *P* = 0.043) remained significant as the independent predictors for all-cause mortality of the patients. (Table 1)

### The interval from the patients leaving endemic areas to onset of penicilliosis

Among the 47 penicilliosis patients, 33 patients were excluded from the interval statistics according to the definition of incubation period, because they already presented with penicilliosis-related symptoms when they were in the endemic areas; 14 patients were included in the interval statistics according to the definition of incubation period, because they presented with penicilliosis-related symptoms after returning to Hubei province which is not among the endemic areas. As shown in the Table 4, 10 (71.4%) patients out of 14 presented with onset of symptoms within 12 months after leaving the endemic areas, of whom 7 patients presented with onset of symptoms within 6 months after leaving the endemic areas, 3 patients presented with onset of symptoms 6–12 months after leaving the endemic areas. Four (28.6%) patients out of 14 presented with onset of symptoms 1–8 years after leaving the endemic areas.

**Table 4:** The interval from 14 patients leaving endemic areas to onset of penicilliosis.

Case number	Sex	Age(years)	Endemic areas	Exposure time in endemic areas (days)	Interval (days)
**1**	Male	33	Guangdong	19	11
**2**	Male	27	Fujian	1095	11
**3**	Male	38	Guangdong	7	21
**4**	Male	40	Guangdong	90	45
**5**	Male	24	Guangdong	3650	46
**6** ^**1**^	Female	25	Guangdong	1825	90
**7**	Male	39	Guangdong	120	160
**8**	Male	23	Hunan	40	200
**9**	Male	41	Guangdong	7	344
**10**	Female	31	Hunan	730	360
**11**	Male	31	Guangdong	2920	510
**12**	Female	23	Guangdong	30	1087
**13**	Male	42	Guangxi	730	1095
**14**	Female	38	Fujian	2190	2920

^1^Patient #6 was an asymptomatic carrier with fungemia of *P*. *marneffei*.

In this study, we found an asymptomatic carrier with culture-confirmed fungemia of *P*. *marneffei* 3 months after her departure from the endemic area. She had not received any antifungal therapy and did not display any penicilliosis-associated symptoms until 18 months’ follow up.

## Discussion

Hubei Province is located in central China, which is not a penicilliosis-endemic area. In this study, we report the clinical features of penicilliosis in a non-endemic area of China. Penicilliosis patients accounted for 4.8% of 981 AIDS-related admissions at Zhongnan hospital in Hubei Province from 2006 to 2013, which was lower compared to the report from the Eighth People's Hospital in Guangzhou City (12.5%, 95/762) which is the endemic areas of penicilliosis in China[10], and also lower compared to the report from the National Hospital for Tropical Diseases in Hanoi City, Vietnam (11.0%, 87/793)[11]. In our study, the median age of 47 patients was 32 years old (IQR: 26–40 years), which might be due to the special background of the immigrate workers and travelers who have been included.

Most penicilliosis patients presented with fever, cough, sputum production, cutaneous lesions and hepatosplenomegaly. In this study, *P*. *marneffei* was isolated from samples of bone marrow, blood, sputum, bronchoavleolar lavage, stool, urine, skin biopsy/scrapings, ascetic fluid, lymph nodes, and ocular humor by microbial cultures, except for samples of cerebrospinal fluid. This suggests that penicilliosis is a systemic disseminated fungal disease, but that *P*. *marneffei* may not have the capability to get through the blood-brain barrier.

The transmission routes of *P*. *marneffei* infection were not clear. A previous study suggested that penicilliosis may be associated with exposure to soil in the rainy season[12]. In this study, most of the penicilliosis patients presented with respiratory symptoms, 59.6% (28/47) of patients had cough, sputum production, and dyspnea; 84.1% (37/44) of patients had abnormal chest CT characteristics. The high positive rate of *P*. *marneffei* from sputum (11/17) and BAL (8/9) cultures suggested that it might be acquired through inhalation from the environment.

Agranulocytosis (neutrophils <0.5×10^9^ cells/L) persisting for more than 10 days is considered to be the major risk factor for acquiring invasive fungal disease in patients with blood disorders, organ transplants and cancers[13]. In our study, only 12 (25.5%) patients out of 47 had a neutropenia (neutrophils <2.0×10^9^ cells/L) and none of these had agranulocytosis, but 44 penicilliosis patients (44/47, 93.6%) had a CD4+ T-cell count ≤50 cells/μL, suggesting that penicilliosis patients often have severe cellular immune dysfunction but uncommonly present with agranulocytosis.

In our study, thirty-four patients out of 47 (72.3%) who were hospitalized due to penicilliosis were then diagnosed with HIV infection during their hospitalization. The CD4+ T-cell counts were very low for these patients, with a median of 8 cells/μL (IQR: 3–21 cells/μL). This supports existing data that describe the difficulty of obtaining early diagnosis of HIV/AIDS in some areas of China, which leads to the delay of cART initiation. There remains much room for improvement in surveillance in the population at high risk for HIV/AIDS, as well as for pre- and post-test counseling.

The median interval from the onset of penicilliosis symptoms to the confirmed diagnosis was 36 days (range:7–212 days) in our study. This reflects a lack of vigilance of the clinicians regarding penicilliosis in non-endemic areas of China, and may be exacerbated by the unavailability of fungal cultures in some primary health care units of the villages. Therefore, in non-endemic areas, when physicians observe AIDS patients who have traveled to endemic areas of penicilliosis and present with certain clinical manifestations such as fever, cutaneous lesions, cough, hepatosplenomegaly, lymphadenopathy, and/or abnormal imaging of chest CT scan, they should consider penicilliosis as part of the differential diagnosis. Since fungal cultures are unavailable in some primary health care units, the travel history may play an important role in the differential diagnosis for penicilliosis and should be investigated carefully.

The fungal culture is a reliable tool for the diagnosis of penicilliosis, but it is not convenient for clinical use or for early diagnosis because *P*. *marneffei* generally takes 3 to 7 days to grow. Currently, there are no commercial kits for the rapid testing of *P*. *marneffei*. However, according to the literature, *P*. *marneffei* may interact with Aspergillus antigen[8]. Yu-Tsung Huang *et al*. conducted a study which detected the serum level of galactomannan, an aspergillus antigen component, in a group of penicilliosis patients, and compared them with a group of patients with cryptococcal meningitis and a negative control group by aspergillus antigen kit, and found that the sensitivity of diagnosis for penicilliosis was 66.7% (10/15) with a specificity of 90.9% (30/33) (cut-off value = 1.0)[14]. We have replicated these results in our 24 penicilliosis patients and 33 AIDS patients without penicilliosis. The data showed that the median GM OD index was 5.354 in the group of penicilliosis patients, compared to 0.422 in the group of AIDS patients without penicilliosis. The sensitivity of diagnosis for penicilliosis by this kit was 95.8% (23/24) and the specificity was 90.9% (30/33), suggesting that there is a cross-reaction between *P*. *marneffei* and Aspergillus antigens. In the 33 patients without penicilliosis, two cryptococcosis patients and 1 tuberculosis patient had positive GM results. Although previous literature has reported that *C*. *neoformans* galactoxylomannan can cross-react with Aspergillus galactomannan[15], co-infection with Aspergillus could not be excluded. Since serum cryptococcal antigen can be detected by the Cryptococcal Latex Agglutination Test System, the GM antigen test does not affect the diagnosis of cryptococcosis in patients with AIDS.

We have compared the time required for diagnosis of penicilliosis between serum GM antigen testing and fungal culture. If serum GM antigen was obtained the day after admission, it would be 6 days earlier than the fungal culture for diagnosis of penicilliosis. Although the serum galactomannan test for diagnosis of penicilliosis is not specific, the incidence of aspergillosis infection is relatively low in AIDS patients (2.8‰, 3/1044)[16]. In our study, only 3 patients were confirmed for aspergillosis by biopsy and culture among 981 AIDS patients (3.1‰), suggesting that in AIDS patients, the serum galactomannan test had utility for the early diagnosis of penicilliosis in the absence of a specific serum diagnostic method. Since it is difficult to differentiate between penicilliosis and some OIs because they have some similar manifestations, (for example, military penicilliosis can easily be misdiagnosed as miliary pulmonary tuberculosis based on radiological findings,) the GM test may play a role in the differentiating between them. In addition, GM antigen levels are very high for patients with penicilliosis before antifungal therapy is initiated and the assay is very sensitive for diagnosis of penicilliosis. Antigen levels may be used to monitor the response to antifungal therapy and to monitor relapse. This is a topic worthy of further study.

Twenty-six (2.7%) patients out of 981 AIDS-related admissions died during the hospitalization. Among them, 3 deaths (3/26, 11.5%) were caused by penicilliosis, which made it the third leading cause of death for AIDS patients in the hospital, just behind cryptococcosis (8/26, 30.8%) and pneumocystosis (6/26, 23.1%). The early all-cause mortality of penicilliosis patients (death within 12 weeks) increased from 6.4% (3/47) to 21.3% (10/47), if we compare early deaths of penicilliosis patients in the hospital with total deaths (inside and outside of the hospital), for a few patients have given up their treatment due to the advanced severe disease and so be discharged from the hospital before death. Overall, our data was comparable to the all-cause mortality of the penicilliosis patients reported at Ho Chi Min City Hospital for Tropical Diseases (19.6%, 101/513)[17]. In a previous study, the reported mortality of untreated penicilliosis was 100.0%[18]; in this study, 6 (14.3%) of 42 patients with antifungal therapy were dead within 12 weeks, and 4 of 5 patients (80.0%) without antifungal therapy were dead within 12 weeks.

As for the 34 patients who survived, 2 (5.9%) of them relapsed due to improper withdrawal of the antifungal therapies when their CD4+ T-cell count reached 46cells/μL and 37cells/μL. Wu TC *et al*. retrospectively surveyed 47 penicilliosis patients, and reported a relapse rate of 4% in 5 years[19].

In our study, multivariate analysis showed the delay of the diagnosis for penicilliosis (OR 1.019, CI 95%:1.000–1.038, *P* = 0.046) and the absence of antifungal treatment (OR 14.178, CI 95%:1.084–185.382, *P* = 0.043) independently predicted the early mortality of the patients. Therefore, the efforts should be done for an early diagnosis and a standardized antifungal treatment in order to improve the prognosis of patients with penicilliosis.

Few studies have reported the incubation period of penicilliosis. Recently, Philip L. Bulterys *et al*. suggested an incubation period of 1 week (95%CI, 0–3 weeks) by maximum likelihood estimation given that the penicilliosis admissions were strongly associated with environmental humidity [20]. In this study, 14 patients presented with the penicilliosis-related symptoms after returning to the Hubei province, which is not an endemic area. The interval from the patients leaving endemic areas to the onset of penicilliosis is shown in the Table 4. Ten (71.4%) patients out of 14 presented with onset of symptoms within 12 months after leaving the endemic areas. However, the incubation time appears to be quite different from one penicilliosis patient to another, from 11 days to as long as 360 days. In addition, 4 patients presented with onset of symptoms 1–8 years after their departure from the endemic areas. The reasons for this late presentation may be due to the fact that the incubation time of penicilliosis depends on the cellular immunity of the host, such as infection by tuberculosis; there are no clinical symptoms until the cellular immune function of the host has been severely impaired. Moreover, in this study, we also found an asymptomatic carrier with fungemia of *P*. *marneffei* who survived without any antifungal therapy. A previous report by Teresa K. F. Wang *et al*, also indicated that there exists a latent form of infection by *P*. *marneffei*[21].

## Conclusions

In this study we provide evidence that co-infection of *P*. *marneffei* and HIV is not uncommon in non-endemic areas of *P*. *marneffei* such as the Hubei Province of China. Penicilliosis patients often have severe cellular immune dysfunction but scarcely present with agranulocytosis. Penicilliosis is a systemic disseminated fungal disease which carries a high mortality especially if the diagnosis is delayed. The incubation period of penicilliosis differs from patient to patient, and there appears to be a latent form of *P*. *marneffei* infection. In AIDS patients, the serum galactomannan test has utility for the diagnosis of penicilliosis. When patients with penicilliosis/AIDS were diagnosed early and treated with standardized antifungal therapy and combined antiretroviral therapy, their prognosis improved.
